# Congenital Stenosis of the Pylorus

**Published:** 1912-03-30

**Authors:** C. Willett Cunnington


### CONGENITAL STENOSIS OF THE PYLORUS.

By C. Willett Cunnington, M.B., B.C.

This condition, although comparatively rare, should be in the minds of general practitioners, who are otherwise liable, should they meet with a case, to mistake it for some less serious condition, with grave results to the patient. The cases one sees sent up for diagnosis to children's hospitals are, not seldom, so advanced that this statement is justified. Probably the disease occurs in general practice more often than is supposed, and accounts for some of the deaths ascribed to "marasmus."

Statistics show that the condition is commoner in male infants and in first-born children. Although named "congenital," the disease seldom appears before the first month after birth nor later than the end of the second month. But it may have remained unrecognised until the baby is as old as four months. Few unrecognised cases would survive beyond that age.

The clinical symptoms presented by a typical case are, in the main, four: Vomiting, constipation, visible peristalsis, and wasting. Without these four symptoms the diagnosis in a given case must be regarded as doubtful. Of the vomiting the important feature is that it is curiously violent in character, so that the stomach contents are shot out, often through the nose as well as through the mouth. Further, the amount vomited will from time to time prove to be greater than that taken at the preceding meal. By itself this feature is very suggestive. The vomiting may not be more frequent than once or twice a day; rarely a whole day passes without its occurrence.

The constipation is such a constant feature that the mere absence of this symptom should suggest some other diagnosis.

The peristalsis is not constantly present, nor always easy to see. It is most likely to be visible during or just after a meal. The wave passes from left to right, and is not to be confused with peristalsis of the transverse colon, which moves in the opposite direction. On palpation a hard nodule, the thickened pylorus, may from time to time be felt. The wasting is an essential feature, and is due of course to the constant vomiting. It may be extreme in cases of some duration. Mere change of diet proves unavailing or of only temporary (and therefore misleading) benefit.

Although eventually the disease develops well-marked features, it is highly necessary that valuable time be not wasted in waiting for the diagnosis to become "writ large." Too often this is allowed to happen, while change after change of diet is tried, producing brief and deceptive improvements; a slight gain in weight is often got by these changes of food, only to be lost again as time goes by. Treatment, to be successful, should be adopted early. We may even say that treatment should precede diagnosis, and of all forms of treatment there is none easier and safer than stomach-washing.

After a few demonstrations the mother can be taught, if need be, to perform this simple manœuvre. A soft No. 12 catheter, with funnel attached, is passed 8 inches down the œsophagus. The child, lying flat, is wrapped firmly in a shawl, so that the arms are secured. In this way an assistant is not required. The stomach is syphoned out with weak bicarbonate of soda solution (two grains to the ounce). This is repeated several times until the washings are clear. Afterwards a feed is poured down the tube and left in the stomach. In bad cases it may be necessary to wash out the stomach two or three times a day; as improvement takes place, once a day and then on alternate days will suffice. The washing out will probably have to be employed for several weeks.

Rectal feeding at the beginning of treatment is sometimes recommended, but it has the great drawback that but little nourishment is absorbed, and in bad cases we cannot afford to keep the infant in a state of semi-starvation such as obtains when rectal feeding alone is employed.

An instrument only second in importance to the stomach tube is the weighing machine. This must be of an accurate kind and capable of measuring to half-ounces. I have myself learned to distrust all forms of spring balance in weighing infants. The resilience of a spring varies so much with changes of temperature that such a form of weighing machine is most deceptive. At present a cheap yet reliable balance is not to be obtained.

The weight of the patient should be recorded at least twice a week and always under similar conditions. This is essential, for the changes in weight form the important index in treatment of these cases. An immediate gain in weight is not to be expected, after starting the washing-out treatment. At first it is sufficient if the decrease is checked by it. After a week or two a slight gain will be observed; gradually the increment increases, but we must be satisfied with slow progress, as long as it is in the right direction.

As regards the nature of the food, there can be no question that breast-milk, if available, is best. If necessary it may be drawn off by pump and introduced into the infant's stomach *viâ* the tube. If breast milk is not available we are driven to use cow's milk modified either by dilution alone or by peptonisation, or even by removal of its casein—in other words, by giving whey. Peptonised milk appeals to the mind as being already partly digested, but in practice one only too often finds that on it an infant refuses to increase in weight to the extent it should theoretically. A more satisfactory food is obtained by a mixture of whey, raw meat juice, and cream. Later on, cow's milk can be gradually introduced, especially if sodium citrate (gr. ij ad ℥j) be added to ensure the casein curds being precipitated in a fine state.

The amount of each feed is an important matter. A rough guide is to give half the ordinary quantity for the age at half the ordinary time-intervals. Immediately after each meal the infant is to be laid down and kept as quiet as possible lest vomiting ensue. For the constipation phenol-phthalein in doses of $\frac{1}{2}$ grain to 1 grain is a convenient drug.

As regards the value of surgical treatment for this condition, opinions vary. An opinion based on statistics is necessarily misleading, the surgeon complaining that his failures are in the bad cases which have been allowed to remain until they are *in extremis*; on the other hand, the physician will argue that the milder cases do not need operation, as they respond well, as a rule, to the medical treatment such as has been described.

Gastro-enterostomy, forcible dilatation of the pylorus, and pyloroplasty each have their advocates; at present the last-named operation is usually chosen. The pylorus is exposed, a small longitudinal incision is made which is then sewn up so as to convert it into a transverse one; in this way the pylorus is shortened and widened. But beyond the immediate risk of such operation upon a small infant, already wasted and feeble, is the danger of persistent disturbance of digestive functions set up by the operation.

